# Improving the In Vitro Bioaccessibility of β-Carotene Using Pectin Added Nanoemulsions

**DOI:** 10.3390/foods9040447

**Published:** 2020-04-07

**Authors:** Júlia Teixé-Roig, Gemma Oms-Oliu, Sara Ballesté-Muñoz, Isabel Odriozola-Serrano, Olga Martín-Belloso

**Affiliations:** Department of Food Technology, University of Lleida-Agrotecnio Center, Av. Rovira Roure 191, 25198 Lleida, Spain; julia.teixe@udl.cat (J.T.-R.); gemma.oms@udl.cat (G.O.-O.); sarabm.albatarrec@gmail.com (S.B.-M.); isabel.odriozola@udl.cat (I.O.-S.)

**Keywords:** β-carotene, nanoemulsions, pectin, stability, bioaccessibility

## Abstract

The intestinal absorption of lipophilic compounds such as β-carotene has been reported to increase when they are incorporated in emulsion-based delivery systems. Moreover, the reduction of emulsions particle size and the addition of biopolymers in the systems seems to play an important role in the emulsion properties but also in their behavior under gastrointestinal conditions and the absorption of the encapsulated compound in the intestine. Hence, the present study aimed to evaluate the effect of pectin addition (0%, 1%, and 2%) on the physicochemical stability of oil-in-water nanoemulsions containing β-carotene during 35 days at 4 °C, the oil digestibility and the compound bioaccessibility. The results showed that nanoemulsions presented greater stability and lower β-carotene degradation over time in comparison with coarse emulsion, which was further reduced with the addition of pectin. Moreover, nanoemulsions presented a faster digestibility irrespective of the pectin concentration used and a higher β-carotene bioaccessibility as the pectin concentration increased, being the maximum of ≈36% in nanoemulsion with 2% of pectin. These results highlight the potential of adding pectin to β-carotene nanoemulsions to enhance their functionality by efficiently preventing the compound degradation and increasing the in vitro bioaccessibility.

## 1. Introduction

Carotenoids are important compounds that act as natural pigments and have been related to several potential health benefits as the prevention of some cancers, cardiovascular diseases, macular degeneration, or cataracts [1,2,3]. β-carotene is among the main carotenoids present in the human diet [4] and it provides the highest provitamin A activity [5]. Nevertheless, the biological activity of β-carotene is highly dependent on its intestinal absorption, which is often inefficient as a consequence of entrapment in food matrices, and low stability under gastrointestinal conditions, among others [6,7,8]. Besides, the application of β-carotene in many food matrices is limited because the compound is poorly dispersed in water and its chemical stability is low, being specially sensitive to heat, oxidation, and light due their unsaturated chemical structures [9,10].

Emulsion-based delivery systems can be produced using various emulsification processes and they have been widely used to protect bioactive compounds such as carotenoids from degradation, ameliorate its dispersion in an aqueous media, and increase its bioaccessibility [10,11,12,13,14,15,16]. However, in the last years, oil droplet reduction in the range of 20–500 nm [17] showed several advantages over conventional emulsions. Nanoemulsion-based delivery systems have been reported to be more resistant to gravitational separation and aggregation than conventional emulsions [18,19]. Moreover, their high surface area seems to facilitate the digestive enzyme activity [20,21] and increase the compound bioaccessibility [10,22,23,24,25].

Pectin is a naturally-sourced biopolymer composed of a group of complex polysaccharides rich in galacturonic acid units linked by α (1–4) bonds located in the cell wall of plants. This molecule has an amphiphilic character that helps to reduce the interfacial tension between oil and water phases, providing good emulsification properties [26,27], and also increases the viscosity of the aqueous phase [28]. Actually, consumers are interested in more natural products, so the use of natural ingredients in the formulation of delivery systems is an actual area of interest. In that sense, pectin has been used as an emulsifying and stabilizing agent in food emulsions, but although the use of this biopolymer together with other emulsifiers and their possible interactions has been studied in emulsions, there is a lack of knowledge on its effect on nanoemulsions and, specifically on its behavior under gastrointestinal conditions. Moreover, the addition of biopolymers such as pectin in emulsion-based delivery systems not only can modify the initial emulsion characteristics, but also the behavior of the systems under gastrointestinal conditions and the bioaccessibility of the encapsulated compound [29,30,31,32,33,34,35]. Thus, pectin can have an impact on some health important factors as the satiety, the glycaemia control, and the prevention of some gastrointestinal diseases [36]. Hence, the present study aimed to evaluate the effect of pectin addition (0%, 1%, and 2%) on the physicochemical stability of oil-in-water nanoemulsions containing β-carotene during 35 days at 4 °C, the oil digestibility and the compound bioaccessibility.

## 2. Materials and Methods

### 2.1. Materials

β-carotene, Tween 20, pepsin (from porcine gastric mucosa), pancreatin (from porcine pancreas), bile extract (bovine), and all the solvents were obtained from Sigma-Aldrich, Inc. (St. Louis, MO, USA). Corn oil was purchased from a local supermarket. Food-grade high methoxyl pectin from citrus peel with a degree of methylesterification from 67% to 71% was obtained from Acros Organics (Morris Plains, NJ, USA). Ultrapure water, obtained from Millipore Milli-Q filtration system water was used to prepare emulsions and reagents of the experiment.

### 2.2. Methods

#### 2.2.1. Coarse Emulsion and Nanoemulsions Preparation

To obtain the lipid phase, β-carotene was dissolved in corn oil (5 mg·g^−1^) by sonicating (1 min) and stirring (45 °C, 5 min). To formulate the aqueous phase, pectin (0%, 1%, and 2%) was added into ultrapure water, previously heated at 70 °C, and dispersed using an homogenizer (Ultra-Turrax, Janke & Kunkel, Staufen, Germany) at 9500 rpm for 5 min. The aqueous phase (containing water and pectin) was left for 1 h until it was at room temperature. Then, the lipid phase (4% *w*/*w*), Tween 20 (4% *w*/*w*) and the aqueous phase were homogenized at 9500 rpm for 2 min to obtain the coarse emulsion. Finally, a microfluidizer (M-110P, Microfluidics, Newton, MA, USA), equipped with a 75 µm ceramic interaction chamber (F20Y) at an operational pressure of 100 mPa, was used to form nanoemulsions by passing the coarse emulsion for 5 cycles.

#### 2.2.2. Particle Size

Particle size of emulsions was measured using a Mastersizer 3000 (Malvern Instruments Ltd., Worcestershire, UK). Samples were diluted in ultrapure water and stirred in the dispersion unit at a constant speed of 1800 rpm. The particle size was expressed as surface area mean diameter (*d*_32_) in nanometers (nm), fixing a refractive index of the corn oil of 1.473 and 1.333 for water. Moreover, due to the presence of large particles in some samples, *d_90_* was also reported.

#### 2.2.3. Electrical Charge

The electrical charge (ζ-potential) was measured by phase-analysis light scattering (PALS) using a Zetasizer NanoZS (Malvern Instruments Ltd. Worcestershire, UK) to determine the surface charge at the interface of the droplets. Emulsions were diluted (1:10) in ultrapure water and placed in a capillary cell equipped with two electrodes to assess the electrophoretic mobility of the particles. The results were reported in millivolts (mV).

#### 2.2.4. Stability

The stability of emulsions was studied using an optical scan analyzer Turbiscan MA 2000 (Formulaction, Toulouse, France) which is a non-destructive method that can measure the static stability of samples and detect the cause of instability (flocculation, coalescence, sedimentation, or creaming) by the multiple light scattering technique. A sample of 7 mL was introduced into a glass cylindrical cell and analyzed by a light beam emitted in near infrared wavelength, which scanned vertically from the bottom to the top of the sample cell. Two synchronous optical sensors received light backscattered by the sample (45° from the incident radiation). In this study, the backscattering was measured during 35 days at 4 °C to assess the stability of emulsions over time. The backscattering was analyzed at three different zones of the test tube (top, middle, and bottom) in order to study different instability phenomena throughout the tube such as creaming at the top, flocculation, or coalescence in the middle and sedimentation at the bottom.

#### 2.2.5. β-Carotene Extraction and Quantification

β-carotene determination was carried out according to a previously reported method with some modifications [37]. To extract the β-carotene, 5 mL of sample was mixed with 5 mL of chloroform, vortexed and centrifuged at 1750 rpm for 20 min at 4 °C. After centrifugation, the bottom chloroform phase (orange colored) was collected, while the top layer was vortexed with another 5 mL of chloroform and centrifuged at 1750 rpm at 4 °C during 20 min. The bottom layer, which contained β-carotene, was collected, while the top layer was mixed with an additional 5 mL of chloroform and the same procedure was repeated. Then, the absorbance of the collected bottom layers was measured at 450 nm using a UV-visible spectrometer (CECIL CE 2021; Cecil Instruments Ltd., Cambridge, UK), using chloroform as a blank. The β-carotene content from a sample was measured from a previously prepared calibration curve of absorbance versus β-carotene concentration in chloroform.

#### 2.2.6. In Vitro Digestion

To simulate the human digestion process, an in vitro gastrointestinal tract (GIT) digestion based on an international consensus method [38] with some modifications was used. The coarse emulsion and nanoemulsions were digested immediately after their preparation.

The protocol included both gastric and small intestinal phases. Briefly, 20 mL of the sample was mixed with 18.2 mL of simulated gastric fluid (SGF) containing pepsin (2000 U·mL^−1^), 0.4 mL HCl solution (1 M), and 10 μL of a CaCl_2_ solution (0.3 M). Finally, 1.39 mL of ultrapure water was added to reach a final volume of 40 mL. The mixture was placed into an incubator at 37 °C for 2 h while shaking at 100 rpm. To simulate the intestinal phase, a pH-stat device was used. Once the gastric phase was completed, an aliquot of 30 mL of gastric sample was placed in a 37 °C water bath. Then, 3.5 mL of bile solution (54 mg·mL^−1^) and 1.5 mL of salt solution (NaCl 0.150 mM and CaCl_2_ 0.01 mM) were added and the pH was adjusted to 7 with NaOH (1 M). Finally, 2.5 mL of pancreatin solution (75 mg·mL^−1^) was incorporated into the mixture. The pH of the sample was maintained to 7 by adding NaOH (0.25 M) constantly for 2 h. The final volume of NaOH (0.25 M) was recorded and used to calculate the amount of free fatty acids (FFAs) released during the intestinal phase. The FFA (%) was determined according to Equation (1):

(1) $$FFA\left(\%\right)=\frac{V_{NaOH}{\times \;C}_{NaOH}{\;\times M}_{oil}}{{2\;\times \;m}_{oil}}\;\times 100,$$

where *V_NaOH_* is NaOH volume (L) used during the intestinal digestion, *C_NaOH_* is NaOH molarity (0.25 mol·L^−1^), *M_oil_* is corn oil molecular weight (800 g·mol^−1^), and *m_oil_* is corn oil total weight present in the emulsions (g).

#### 2.2.7. Bioaccessibility Determination

Aliquots of digested emulsions were centrifuged at 4000 rpm during 40 min at 4 °C [39] to obtain the micellar fraction. The concentration of β-carotene in the micellar fraction was determined following the method described in Section 2.2.5. Lastly, β-carotene bioaccessibility was calculated according to Equation (2):

(2) $$Bioaccessibility\left(\%\right)=\frac{C_{micelle}}{C_{initial}}\;\times 100,$$

where *C_micelle_* is the β-carotene concentration (mg·ml^−1^) in the micellar fraction and *C_initial_* the initial β-carotene concentration (mg·ml^−1^) in the emulsion.

#### 2.2.8. Optical Microscopy

Images of coarse emulsion and nanoemulsions were obtained using an optical microscope (Olympus BX41, Olympus America Inc., Melville, NY, USA) with a 100× objective lens. The images were obtained using a digital camera (Olympus DP74) and processed with the software CellSens (Olympus).

#### 2.2.9. Statistical Analysis

All experiments were assayed in duplicate and three repetitions of each analysis were carried out on each parameter in order to obtain mean values. Analysis of the variance (ANOVA) was performed to compare treatments. Least significant difference (LSD) test was employed to determine differences between means. The confidence interval was set at 0.95 and all results were analyzed using the Statgraphics Plus v.5.1 Windows package (Statistical Graphics Co., Rockville, MD, USA).

## 3. Results and Discussion

### 3.1. Physicochemical Characterization

#### 3.1.1. Particle Size

**Figure 1:**
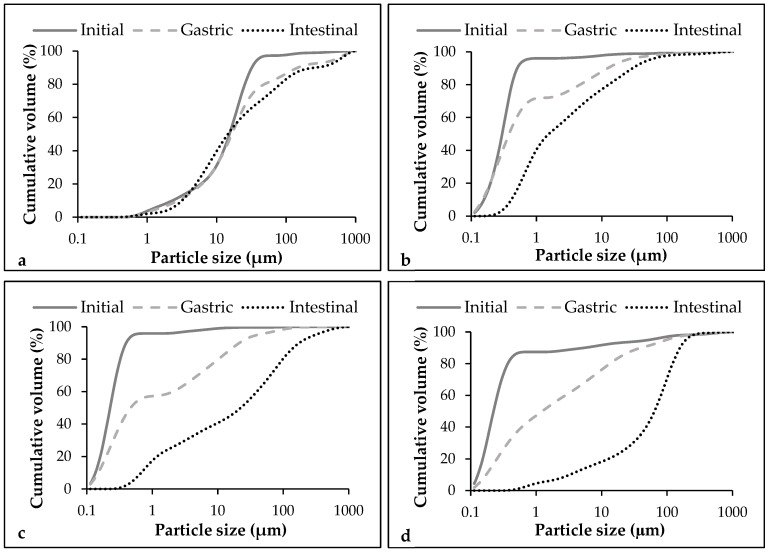
Particle size distribution of coarse emulsion and nanoemulsions with different pectin concentrations (0%, 1%, and 2%) at different phases of the in vitro digestion. (**a**) Coarse emulsion without pectin; (**b**) Nanoemulsion without pectin; (**c**) Nanoemulsion with 1% of pectin; (**d**) Nanoemulsion with 2% of pectin.

Initially, the coarse emulsion presented a higher mean particle size *d_32_* (7300 ± 307 nm) than nanoemulsion without pectin (313.4 ± 20.2 nm) (Table 1). Moreover, *d_90_* value, which indicates the maximum particle diameter of 90% of the sample, showed that the largest droplets in each sample have a size of 25464 ± 1401 nm in the coarse emulsion and 540.7 ± 59.7 nm in the nanoemulsion without pectin. The particle size distribution showed that the microfluidization process not only reduced the mean particle size, but also makes the nanoemulsion less polydisperse compared to the coarse emulsion (Figure 1). A high pressure is applied during the microfluidization process to guide the flow stream through microchannels to the interaction chamber, where cavitation, along with shear and impact, produce a reduction of the emulsion particle size [40]. The addition of pectin significantly (*p* < 0.05) decreased the particle size of nanoemulsions, irrespective of the concentration used. Our results are in accordance with those reported by Verkempinck and co-authors, who also observed a particle size reduction in emulsions that contained a small molecule surfactant (Tween 80) and pectin as stabilizers [41]. On the one hand, the observed decrease in the particle size when pectin was added in the nanoemulsions is suggested to be a consequence of a competitive effect between the polymer and the small molecule surfactant for the interface. Tween 20, which has a lower molecular weight than pectin, may move slightly faster to the interface, leading to small particle sizes [41]. In that sense, we hypothesize that Tween 20 has been efficiently adsorbed in the interface, but also part of the pectin added was present. On the other hand, the viscosity of the nanoemulsions increased from 1.23 ± 0.05 mPa·s in the nanoemulsion without pectin to 6.51 ± 0.10 mPa·s and 19.77 ± 0.26 mPa·s in nanoemulsions with 1% and 2% of pectin, respectively (Table 1). The increase in the viscosity of the continuous phase could enhance the reduction of the particle size by increasing the disruptive shear stresses [42].

#### 3.1.2. Electrical Charge

As it can be observed in Table 1, the electrical charge of the initial coarse emulsion was −32.1 ± 1.9 mV and became less negative after the microfluidization process (−20.9 ± 2.1 mV), as previously reported by other authors [21]. Although the systems are expected to have no negative charge because they are formulated using a non-ionic surfactant (Tween 20), the obtained negative charges can be a consequence of a preferential absorption of OH- species from water to the oil-water interface [43,44]. The ζ-potential became less negative when pectin was added, irrespective of the concentration used (Table 1). This anionic biopolymer has been reported to show negative charges when it is added in emulsions [41,45] due to the carboxyl groups present on its molecule [46], but this fact is influenced by the pH. In our study, nanoemulsion without pectin showed a pH about 6.5, but pectin added nanoemulsions presented a low pH (≈3). At this pH, the pectin molecules lose most of the negative charges because the pKa value of their anionic carboxylic groups is around 3.5, so the majority of carboxyl groups are protonated (-COOH) [28]. Moreover, no variations were observed between nanoemulsions with 1% and 2% of pectin. In that sense, due to the competence for the interface between Tween 20 and pectin, it seems that in both nanoemulsions the same amount of pectin was adsorbed on the interface. Therefore, in the nanoemulsion with 2% of pectin, a high amount of this biopolymer was remaining in the aqueous phase.

### 3.2. Stability

**Figure 2:**
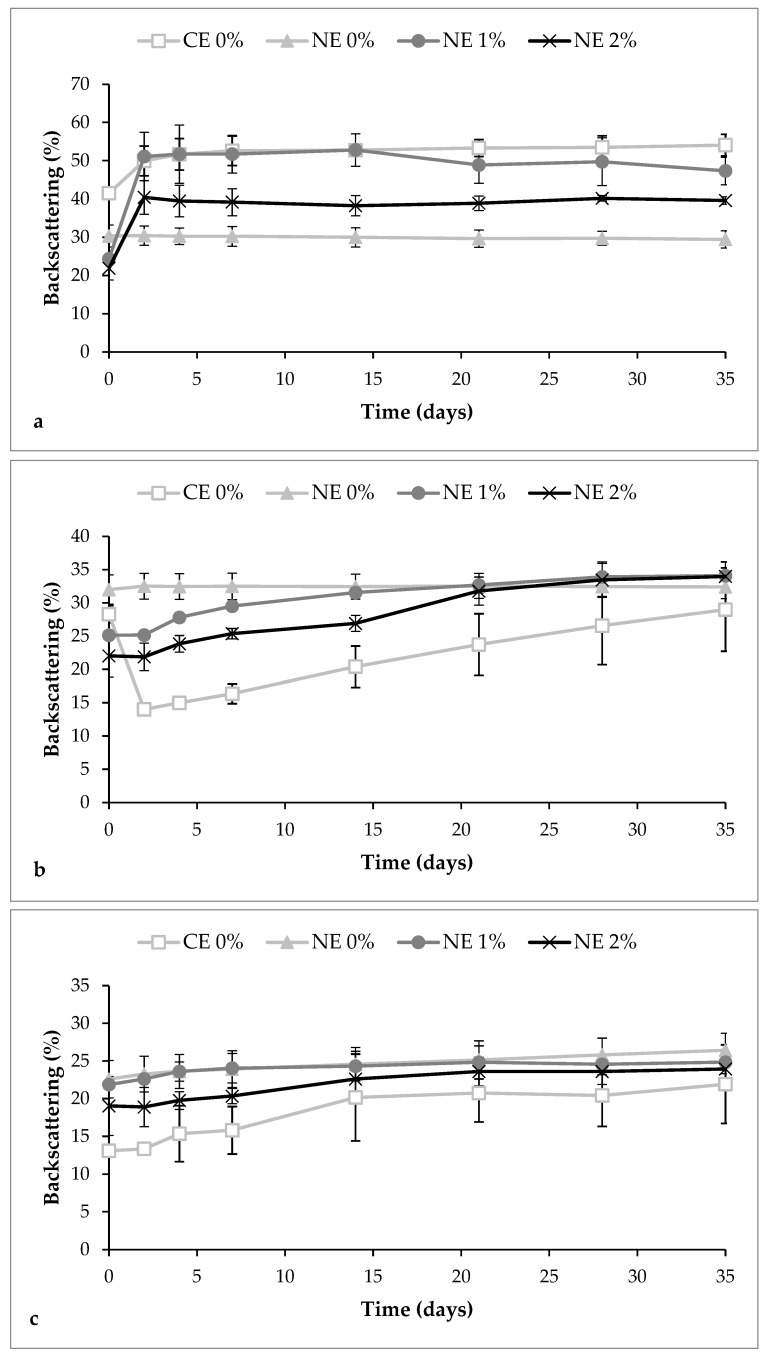
Variation of backscattering values in coarse emulsion and nanoemulsions with different pectin concentrations (0%, 1%, and 2%) at different zones of the test tube during 35 days at 4 °C. (**a**) Top zone; (**b**) Middle zone; (**c**) Bottom zone. CE 0, coarse emulsion without pectin; NE 0 nanoemulsion without pectin; NE 1, nanoemulsion with 1% of pectin; NE 2, nanoemulsion with 2% of pectin.

Initially, the coarse emulsion presented a higher backscattering value at the top zone compared with nanoemulsions as a consequence of the creaming formation in a few minutes after their preparation (Figure 2a). Moreover, in this emulsion, phase separation was observed after 2 days since the oil migrated to the top of the tube, increasing the backscattering at this zone and decreasing it in the middle zone (Figure 2a,b). In contrast, all nanoemulsions exhibited lower variations in backscattering, being the nanoemulsion without pectin the one with least variations (<4%) (Figure 2). Therefore, this nanoemulsion can be considered stable since only variations greater than 10%, either as a positive or as a negative in the graphical scale of backscattering, are considered an indicator of instability [47]. Although both coarse emulsion and nanoemulsion are thermodynamically unstable systems, the small particle size of the nanoemulsion could prevent instability phenomena as sedimentation or creaming since the Brownian motion, and consequently the diffusion rate, are greater than the sedimentation or creaming rate induced by the gravity [48].

**Figure 3:**
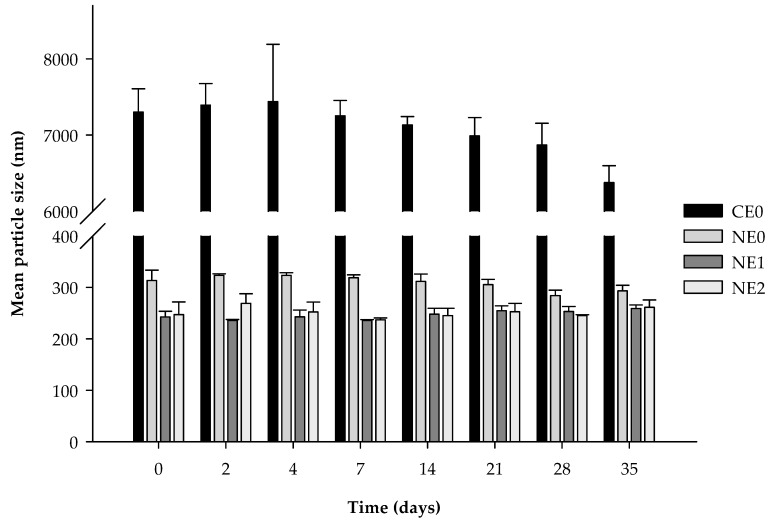
Mean particle size (*d_32_*) of coarse emulsion and nanoemulsions with different pectin concentrations (0%, 1%, and 2%) during 35 days of storage at 4 °C. CE0, coarse emulsion without pectin; NE0 nanoemulsion without pectin; NE1, nanoemulsion with 1% of pectin; NE2, nanoemulsion with 2% of pectin.

The creaming phenomenon was detected in pectin nanoemulsions after 2 days of storage. An increase of 26.8% and 18.5% of the backscattering at the top zone was observed when pectin was added at 1% and 2%, respectively (Figure 2a). Moreover, these nanoemulsions presented an increase of the backscattering in the middle zone after 35 days of storage, more pronounced when the polymer was added at 2% (Figure 2b). This increase in the middle zone is suggested to be a consequence of the reversible flocculation phenomenon rather than coalescence of the droplets since the light scattering measurements did not show an increase of the particle size (Figure 3). Other authors also observed flocculation of droplets, and consequently, the appearance of creaming when citrus pectin was added at concentrations of ≥0.02% in emulsion-based systems [29,45]. When pectin is used at low concentrations, the repulsive interactions between droplets are sufficiently large to overcome the attractive interactions, but there is a critical concentration of the polymer over which the attraction is sufficiently strong to promote flocculation of droplets, and thereby, creaming occurs [32,49]. As previously mentioned, we suggest that the part of pectin that has not been adsorbed at the interface is remaining at the aqueous phase, creating an osmotic imbalance, which promoted the depletion flocculation phenomena [50]. Moreover, the higher amount of flocculation observed in the nanoemulsion with 2% of pectin compared with those with 1%, may be due to the higher amount of biopolymer remaining at the continuous phase.

### 3.3. β-Carotene Degradation

**Figure 4:**
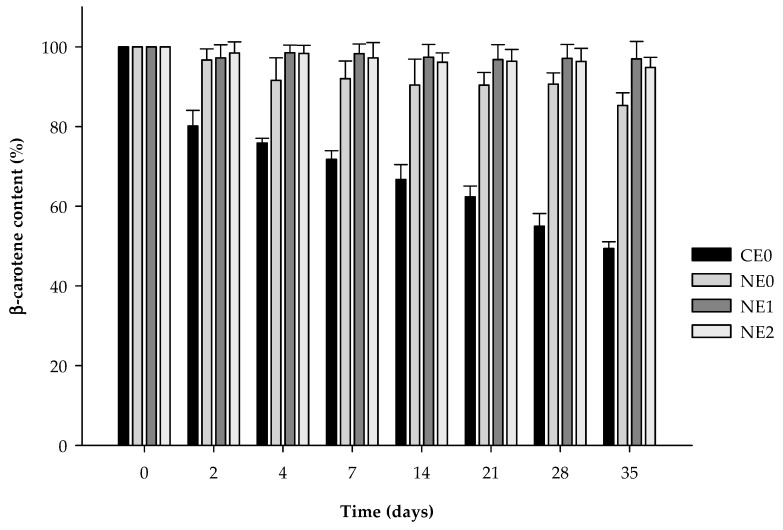
β-carotene content on coarse emulsion and nanoemulsions with different pectin concentrations (0%, 1%, and 2%) during 35 days at 4 °C. CE0, coarse emulsion without pectin; NE0 nanoemulsion without pectin; NE1, nanoemulsion with 1% of pectin; NE2, nanoemulsion with 2% of pectin.

During the storage, the β-carotene content was reduced by 50% in the coarse emulsion, whereas in the nanoemulsion without pectin, the reduction was about 14% (Figure 4). Our results are similar to those reported by other authors who observed degradation of about 14–25% in nanoemulsions formulated with small molecule surfactants, after 4 weeks of storage at 4 °C [7,51]. The reduction of the particle size could increase carotenoid degradation because of a higher surface area exposed to oxidation [51,52]. However, in our study, a rapid phase separation was observed in the coarse emulsion. This phenomenon resulted in a loss of the emulsion structure, which could promote the β-carotene oxidation because it was more exposed to the environment.

The addition of pectin reduced the compound degradation during the 35 days of storage at 4 °C irrespective of the concentration used (Figure 4). At day 35, the nanoemulsion without pectin showed a β-carotene content of 85.3% ± 3.2%, while nanoemulsions with 1% and 2% of pectin presented values of 97.0% ± 4.4% and 94.9% ± 2.6%, respectively. The presence of pectin in the aqueous phase or at the interface may physically hinder the ability of pro-oxidants to interact with β-carotene within the oil droplets by providing a steric barrier [53]. Moreover, pectin has been reported to be effective inhibiting lipid oxidation due to their free radical scavenging activity and their iron-binding capacity [54]. Although pectin nanoemulsions have been observed to present flocculation and creaming (Section 3.2), we suggest that the flocculation phenomenon could be acting as a protective effect by entrapping the compound in the flocs produced by pectin.

### 3.4. Gastrointestinal Behaviour of the Emulsions

#### 3.4.1. Particle Size

**Figure 5:**
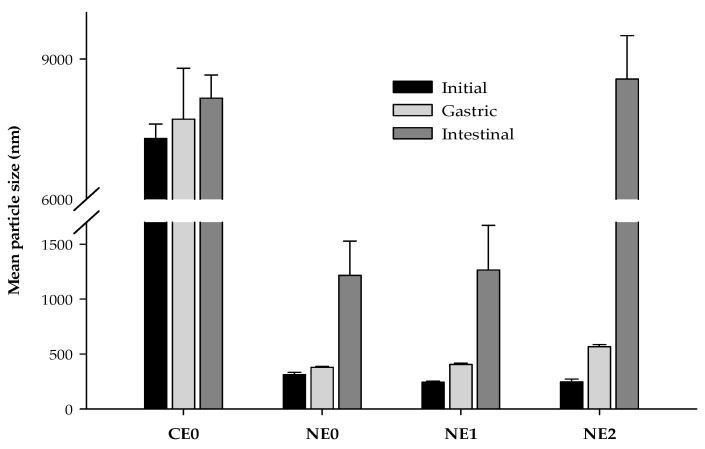
Mean particle size values (*d_32_*) of coarse emulsion and nanoemulsions with different pectin concentrations (0%, 1%, and 2%) at different phases of the in vitro digestion. CE0, coarse emulsion without pectin; NE0, nanoemulsion without pectin; NE1, nanoemulsion with 1% of pectin; NE2, nanoemulsion with 2% of pectin.

**Figure 6:**
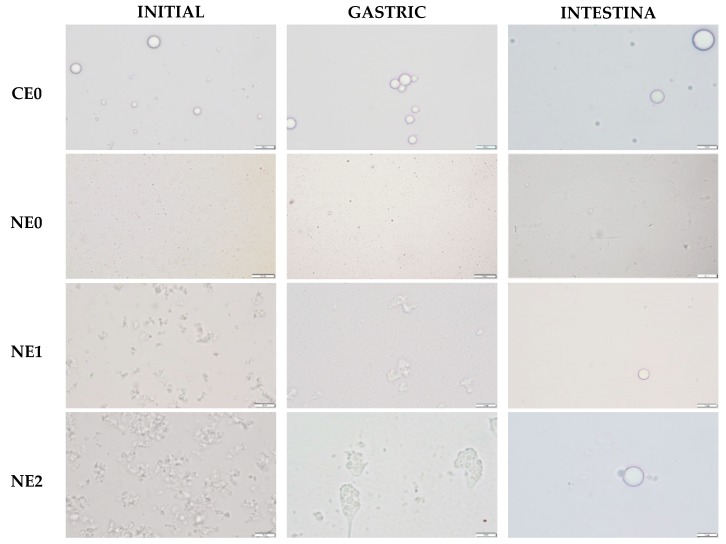
Images of coarse emulsion and nanoemulsions with different pectin concentrations (0%, 1%, and 2%) at different phases of the in vitro digestion. CE0, coarse emulsion without pectin; NE0, nanoemulsion without pectin; NE1, nanoemulsion with 1% of pectin; NE2, nanoemulsion with 2% of pectin. Scale bars were 10 µm long.

In the gastric phase, no significant changes in the mean particle size were observed between coarse emulsion and nanoemulsion without pectin although some changes in particle size distribution were detected (Figure 1 and Figure 5). In both cases, the particle size distribution became more polydisperse, meaning that some large particles were formed (Figure 1). This fact can also be observed in the microscope images of these emulsions in the gastric phase (Figure 6). Few changes on the mean particle size during the gastric digestion were also reported by other authors in oil-in-water emulsions formulated with small surfactants as Tween 20 or Tween 80 [25,55]. This type of surfactants has been reported to provide greater stability under gastric conditions when compared with others like lecithin or proteins, which have shown to be less effective against flocculation under gastric conditions [56,57]. In nanoemulsions with pectin, the mean particle size increased during the gastric phase, more noticeably when the polymer was added at 2% (Figure 5). Moreover, the particle size distribution became more polydisperse, similar to the nanoemulsion without pectin, because of an increase of large particles (Figure 1). The flocculation and coalescence phenomena, which can be observed in the microscope images of pectin nanoemulsions in the gastric phase (Figure 6), could be the reason for the increased particle size at this stage. In that sense, the pectin remaining in the continuous phase of these nanoemulsions may be promoting the formation of aggregates containing both oil droplets and pectin molecules [41]. Moreover, in the microscope images of the nanoemulsion with 2% of pectin (Figure 6), it can be observed that pectin could have induced the formation of gel-like pectin, namely Ca^+2^-crosslinkings with the available calcium ions in the stomach juice, which are clustering oil droplets [14].

During the intestinal phase, the mean particle size increased substantially in the nanoemulsion without pectin, while in the coarse emulsion, no significant variation was detected with respect to the gastric phase (Figure 5). The particle size distribution graphs also showed such an increase in the particle size in the nanoemulsion without pectin (Figure 1). During the lipid digestion, the oil droplets are digested by the intestinal enzymes and the mixed micelles are formed. In that sense, the increase of particle size at this stage could be related to the formation of the mixed micelles, the presence of some other molecules produced in the intestinal digestion or undigested oil droplets. Otherwise, during the intestinal phase, droplet coalescence can also occur, since the release of surface-active products generated during the lipid digestion can displace the interface stabilizers so they are ineffectively preventing the coalescence of oil droplets [16,43]. In nanoemulsions with pectin, larger particle sizes were observed compared with those without the biopolymer, especially in the nanoemulsion with 2% of the pectin (Figure 1 and Figure 5). This fact is confirmed by the microscope images of nanoemulsions with pectin, in which larger particles can be observed (Figure 6).

#### 3.4.2. Electrical Charge

**Figure 7:**
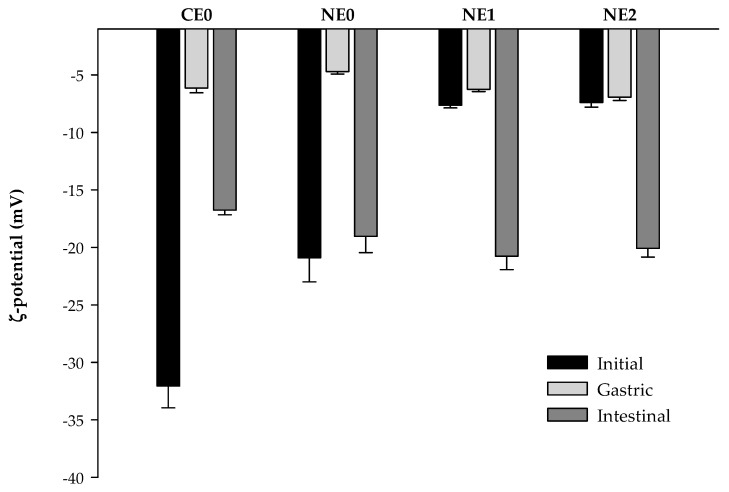
ζ-potential values of coarse emulsion and nanoemulsions with different pectin concentrations (0%, 1%, and 2%) at different phases of the in vitro digestion. CE0, coarse emulsion without pectin; NE0, nanoemulsion without pectin; NE1, nanoemulsion with 1% of pectin; NE2, nanoemulsion with 2% of pectin.

During the gastric phase, the ζ-potential becomes less negative in both coarse emulsion and nanoemulsion without pectin (Figure 7). The electrostatic screening effects resulting from the interactions between oil-water surfaces and ions present at this stage (Ca^2+^, K^+^, and H^+^) could produce a decrease in the negative charge [28,41]. Otherwise, the electrical charge measurements of nanoemulsions with pectin showed that there were little changes under gastric conditions (Figure 5). The initial pH of these nanoemulsions was ≈3, so when they were subjected to the gastric phase, there was no important variation on the pH (2.5). Thus, as well as in the initial systems, the majority of carboxyl groups of pectin were protonated at these conditions, so they cannot interact with the ions present at this stage.

The ζ-potential of emulsions had a relatively high negative charge under simulated intestinal conditions, being more negative in nanoemulsions compared with coarse emulsion (Figure 7). The general increase in the electrical charge (in absolute terms) at this stage could be caused due to the presence of some negatively charged molecules resulting from the intestinal digestion process as bile salts, lipase, or phospholipids that compete with surface-active particles initially present at the droplet surface [43,58,59]. No significant differences were observed between the electrical charge of nanoemulsions with and without pectin at this stage, as reported previously [41]. At neutral pH of the intestine, both the pectin molecule and the lipid droplets have high negative charges, so there could exist a high electrostatic repulsion between them, which may inhibit the pectin adsorption on the interface [60]. Moreover, it is also possible that the high impact of the anionic species resulting from intestinal digestion on the electrical charge was covering up the effect of pectin [57], making it difficult to be observed in the electrical charge measurements.

#### 3.4.3. Oil Digestibility

**Figure 8:**
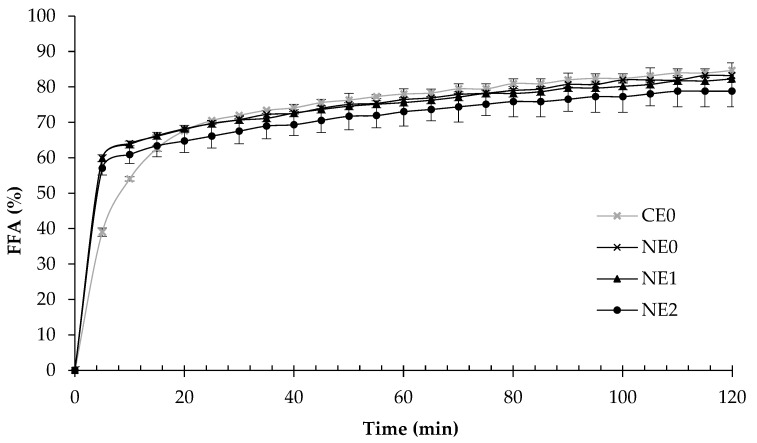
Free fatty acid (FFA) release during the intestinal phase of coarse emulsion and nanoemulsions with different pectin concentrations (0%, 1%, and 2%). CE0, coarse emulsion without pectin; NE0, nanoemulsion without pectin; NE1, nanoemulsion with 1% of pectin; NE2, nanoemulsion with 2% of pectin.

As it can be seen in Figure 8, all emulsions presented the same oil digestibility (about 80%) after the 2 h of the intestinal digestion and the lipid digestion profile presented a similar trend. The amount of FFAs increased rapidly at the beginning of the lipid digestion but slightly at longer digestion times. This suggests that the lipids initially present in the oil droplets were rapidly converted into monoacylglycerols and FFAs. Similar profiles have been previously observed when corn oil has been used in the formulation of emulsions and nanoemulsions [28,39,61]. But looking at the results more specifically, in our study, the initial digestion rate in the coarse emulsion was slower in comparison to all nanoemulsions (Figure 8). In fact, the FFAs liberated in the intestinal phase after 5 min were ≈60% in nanoemulsions and ≈40% in the course emulsion. These differences could be attributed to the particle size of emulsions at the end of the gastric phase. In nanoemulsions, the small particle sizes increase the lipid surface area exposed to lipase activity, enhancing the FFAs release during the intestinal digestion [24,34,62]. Moreover, the access to lipase could be easier in nanoemulsions because they present a thinner layer at the interface due to the higher surface area and same surfactant concentration. This fact could provide less steric hindrance, improving lipase access to oil [63].

The results of this study show that the presence of pectin did not have a significant impact on the rate of lipid digestion of these systems, similar to other published results [60]. As it was previously mentioned, pectin may not be adsorbed at the intestinal pH (pH 7), whereby not affecting the lipolysis process. Other anionic biopolymers as fucoidan had also shown no effect on the lipid digestibility when they are used in combination with small molecule surfactants [57].

#### 3.4.4. β-Carotene Bioaccessibility

The bioaccessibility of a lipophilic compound can be defined as the fraction of the substance which is solubilized within the gastrointestinal intestinal fluids in a suitable form for absorption [20]. β-carotene bioaccessibility increased from 20.9% ± 1.4% in coarse emulsion to 25.0% ± 2.4% in the nanoemulsion without pectin (Table 1). Generally, smaller particle size, i.e., higher surface area, improves the oil digestibility and transfer of β-carotene to micelles [63]. Nevertheless, in our study, no differences were detected between the digestibility of the nanoemulsion and the coarse emulsion. In that sense, although both emulsions presented the same FFAs at the end of the lipid digestion, the faster initial FFAs release in nanoemulsions could have favored the incorporation of β-carotene to the mixed micelles.

The pectin addition affected the bioaccessibility of β-carotene. The higher the pectin concentration, the greater the bioaccessibility, reaching maximum values of 36.9% ± 2.2% in nanoemulsions with 2% of pectin. A similar effect has been observed using mandarin fiber, rich in pectin, on the bioaccessibility of β-carotene nanoemulsions at concentrations up to 1% [34]. In our study, we hypothesize that the flocculation phenomenon in the gastric phase and the formation of pectin gels induced by the presence of pectin in the aqueous phase could act as a steric barrier, reducing the compound degradation during its pass through the gastrointestinal tract and enhancing the β-carotene bioaccessibility. Indeed, the highest bioaccessibility observed in the nanoemulsion with 2% of pectin must be due to the greater flocculation and formation of gels in the gastric phase, as a consequence of a higher amount of pectin present in the aqueous phase. Therefore, although all nanoemulsions exhibited the same amount of free fatty acids at the end of the intestinal digestion, it seems that other factors influenced the bioaccessibility of β-carotene. Some authors have noted that lipid availability for mixed micelle formation is not always correlated with the bioaccessibility of β-carotene [64]. In this regard, factors such as the composition of active molecules in the micelle appear to have also a significant effect.

## 4. Conclusions

The results show that by adding pectin at 2% in nanoemulsions, the lowest β-carotene degradation and the highest bioaccessibility were observed. These results could be related to the ability of this biopolymer to protect the compound by providing a steric barrier and due to its free radical scavenging activity. The reduction in particle significantly increased the stability of the systems, which was probably the cause of the decreased β-carotene degradation observed in the nanoemulsions during the storage. Moreover, lipid digestion was faster in the nanoemulsions, which could produce a faster incorporation of β-carotene in the mixed micelles and, thus, higher bioaccessibility values compared to the coarse emulsion. However, further in vivo investigations are needed to better understand the digestive behavior of pectin nanoemulsions containing β-carotene and to assess the encapsulated compound bioavailability. 

## Figures and Tables

**Table 1:** Particle size, viscosity, ζ-potential, and β-carotene bioaccessibility of coarse emulsion and nanoemulsions with different pectin concentrations (0%, 1%, and 2%). Values are expressed as mean ± standard deviation. Different capital letters indicate significant (*p* < 0.05) differences between the coarse emulsion and nanoemulsion without pectin. Different lower case letters indicate significant differences (*p* < 0.05) between nanoemulsions with different pectin concentrations.

	CE0 ^1^	NE0 ^2^	NE1 ^3^	NE2 ^4^
**Particle diameter *d_32_* (nm)**	7300 ± 307 B	313.4 ± 20.2 Ab	242.6 ± 10.9 a	247.1 ± 24.6 a
**Particle diameter *d_90_* (nm)**	25464 ± 1401 B	540.7 ± 59.7 Ab	448.4 ± 21.4 a	439.3 ± 23.3 a
**Viscosity (mPa·s)**	1.31 ± 0.02 A	1.23 ± 0.05 Aa	6.51 ± 0.10 b	19.77 ± 0.26 c
**ζ-potential (mV)**	−32.1 ± 1.9 A	−20.9 ± 2.1 Ba	−7.6 ± 0.2 b	−7.4 ± 0.4 b
**β-carotene bioaccessibility (%)**	20.9 ± 1.4 A	25.0 ± 2.4 Ba	29.5 ± 1.7 b	36.9 ± 2.2 c

^1^ CE0: coarse emulsion without pectin. ^2^ NE0: nanoemulsion without pectin. ^3^ NE1: nanoemulsion with 1% of pectin. ^4^ NE2: nanoemulsion with 2% of pectin.
